# Obstetricians’ attitudes towards instrumental vaginal delivery in Egypt

**DOI:** 10.1186/s12884-026-09077-2

**Published:** 2026-04-30

**Authors:** Omima T. Taha, Mostafa A. Hamdy, Amal A. Ahmed, Tamer Yehia M. Ali, Mohamed F. Ibrahim, Asmaa M. Elgedawy

**Affiliations:** Department of Obstetrics and Gynecology, Faculty of Medicine, Suez Canal University, Ismailia, Egypt

**Keywords:** Instrumental delivery, Attitude, Obstetricians, Vaginal delivery

## Abstract

**Background:**

Instrumental delivery is a dying art, that needs proper training and practice to revive it and help decrease cesarean delivery rates. This study aimed to declare the obstetricians’ attitude towards instrumental delivery and to elaborate the its possible causes.

**Methods:**

A descriptive cross- sectional study was conducted via an online survey between January 1and April 1, 2025. It was distributed to obstetricians in Egypt. The questionnaire included two main sectors: sector (one) inquired about the sociodemographic characteristics of the participants; sector (two) included the physicians’ attitudes and practices towards instrumental delivery in Egypt.

**Results:**

Seventy-three (52.5%) of the obstetricians never tried ID before. Eighty-nine (64%) of the obstetricians declined encouraging ID if in a situation that indicated the use of ID. Possible reasons for abandoning ID included reduction of training (116/139, 83.5%), lack of senior supervision (99/139, 71.2%), fear of litigation (125/139, 89.9%), lack of consultant supervision in acute situations (108/139, 77.7%), and lack of modern equipment (108/139, 77.7%).

**Conclusion:**

Instrumental delivery is not widely practiced in Egypt. A great proportion of the obstetricians declined to perform/try instrumental delivery in urgent situations.

## Background

Instrumental delivery (ID), also called assisted vaginal delivery, is considered one of six essential skills in basic obstetric emergencies [1]. It has been applied as a management option for obstructed or prolonged labor, maternal exhaustion, poor maternal pushing with epidural anesthesia, or soft tissue obstruction [2]. Recent reports mentioned declined rates of ID in most countries [3]. Rate of ID in Egypt was 2%, while the rates of cesarean section (CS) increased to 52% [4]. A previous study reported that 49% of the Egyptian obstetricians declined to try ID, without exploring possible causes. It also mentioned that the majority of Egyptian obstetricians declined to perform any procedure that would decrease CS rates including ID [5]. Scarce studies reported on the rates of forceps or vacuum delivery in Egypt. One study reported the use of forceps in 8/200 (4%) and vacuum 4/200 (2%) of primiparous women who delivered vaginally [6].

The rate of ID is influenced by many factors as the experience of the operator, the clinical situation, and the availability of these tools [7]. Other reported factors included old/lack of instruments, and restricted training to the old physicians [3]. In a study performed in Latin America, forceps delivery rates were far below those for cesarean delivery, with a continued decline in forceps use over time [8]. Another survey including African countries, vacuum extraction was noted to be superior than forceps, while ID rates remained low and ranged from 1 to 3% [9]. Higher rates of ID delivery were observed in the United States, Canada, and Australia [10]. However; there has been a noticeable decline in ID rates over the last two decades [11]. Possible reasons for declined ID rates, is the rejection of the procedure by both obstetricians and pediatricians. Additionally, cesarean delivery is more feasible and acceptable [12], which lead to increased cesarean delivery rates in Egypt [4]. other reported causes included reluctance of trained physicians to perform ID, lack of training for the younger ones, some countries do not endorse ID use, lack of modern tools, financial gains associated with cesarean delivery, and convinced mothers about the increased safety with cesarean delivery rather than ID [8]. This study was conducted to determine possible causes associated with declined use of ID among obstetricians in Egypt.

## Methods

A descriptive cross- sectional study was conducted via an online survey between January 1and April 1, 2025. The survey was directed to obstetricians in Egypt and the sample was collected as a convenience sampling.

After literature review [13], an English questionnaire was constructed and distributed electronically (via emails and social groups) to the participants for collection of their responses. The questionnaire was tested on 10 participants and then published and distributed electronically. The questionnaire was then validated statistically through principal component analysis and the calculation of Cronbach’s alpha for the whole questionnaire and for each section.

The questionnaire included two main sectors: sector 1 inquired about the sociodemographic characteristics of the participants; sector 2 included the physicians’ attitudes and practices towards instrumental delivery in Egypt. This included either encouraging or refusing practicing instrumental delivery, and possible reasons for their attitude. A panel of three expert obstetricians (professors) revised the questionnaire and determined whether the included items were comprehensive, understandable, clear, and suitable to achieve the aim of the study.

The primary outcome measure was the physicians’ attitude towards ID in Egypt. Secondary outcome measure included possible reasons for avoiding ID among Egyptian obstetricians.

The sample size was calculated at a significance level of 95%and an error level of 20% with the proportion of physicians accepted trial of instrumental delivery in theatre = 60% [14]. A drop-out proportion of 10% was added to the raw result giving a final count of 95 physicians.

### Statistical analysis

Data were fed to the computer and analyzed using IBM SPSS software package version 26.0. **(**Armonk, NY: IBM Corp**)**. Categorical data were represented as numbers and percentages. Quantitative data were expressed as mean and standard deviation.

## Results

The questionnaire was left open for 3 months, collecting 139 responses. It was distributed electronically to a number of 423 obstetricians, with 139 obstetricians responded. The mean age of the responders was 40.42 ± 9.96. A greater proportion of them were females (89/139, 64%) and specialists (63/139, 45.3%). The mean length of experience was 13.91 ± 9.84 years (Table 1).

**Table 1:** Basic demographic data of the participants

Age (years) (Mean ± SD)		40.42 ± 9.96
Sex	Female	89 (64.0%)
	Male	50 (36.0%)
Status	Consultant	54 (38.8%)
	Specialist	63 (45.3%)
	Resident	22 (15.8%)
Residency training	Governmental hospital	87 (62.6%)
	Private hospital	6 (4.3%)
	University hospital	46 (33.1%)
Current practice	Governmental hospital	30 (21.6%)
	Private hospital	28 (20.1%)
	University hospital	21 (15.1%)
	Governmental and private hospitals	39 (28.1%)
	University and private hospitals	21 (15.1%)
Years of experience (years) (Mean ± SD)		13.91 ± 9.84

Seventy-three (52.5%) of the obstetricians never tried ID before. Even those who practiced ID (45/139, 32.4%), had less experience, as they practiced it for few times (1–5 times) (Table 2).

**Table 2:** Physicians’ experience regarding ID

Have you ever tried instrumental delivery	Yes	66 (47.5%)
	No	73 (52.5%)
If yes, how many times	0 times	73 (52.5%)
	1–5 times	45 32.4%)
	6– 15 times	10 (7.2%)
	> 15 times	11 (7.9%)

A great proportion of the physicians had a neutral attitude towards encouraging ID (59/139, 42.4%), although agreeing to present it as an option (64/139, 46%). They presented average knowledge about ID indications and safety (Tables 3 and 4).

**Table 3:** Physicians’ knowledge about ID

ID should be actively encouraged	Agree or strongly agree	35 (25.2%)
	Neither agree nor disagree	59 (42.4%)
	Disagree or strongly disagree	45 (32.4%)
ID should be presented as an option	Agree or strongly agree	64 (46.0%)
	Neither agree nor disagree	46 (33.1%)
	Disagree or strongly disagree	29 (20.9%)
ID should be Offered when there is excessive maternal weight gain, high BMI or gestational diabetes	Agree or strongly agree	18 (12.9%)
	Neither agree nor disagree	30 (21.6%)
	Disagree or strongly disagree	91 (65.5%)
ID should be Offered when the fetal head is more than one-fifth palpable above the pelvic brim	Agree or strongly agree	21 (15.1%)
	Neither agree nor disagree	32 (23.0%)
	Disagree or strongly disagree	86 (61.9%)
ID should be Offered with maternal exhaustion in the second stage	Agree or strongly agree	92 (66.2%)
	Neither agree nor disagree	20 (14.4%)
	Disagree or strongly disagree	27 (19.4%)
ID should be Offered if the fetal head is in the occipitoposterior position	Agree or strongly agree	55 (39.6%)
	Neither agree nor disagree	38 (27.3%)
	Disagree or strongly disagree	46 (33.1%)
ID should be Offered with Fetal skull moulding +++	Agree or strongly agree	22 (15.8%)
	Neither agree nor disagree	37 (26.6%)
	Disagree or strongly disagree	80 (57.6%)

**Table 4:** Physicians’ knowledge about ID

The risk of shoulder dystocia increases with instrumental delivery	Yes	71 (51.1%)
	No	19 (13.7%)
	Unsure	49 (35.3%)
Sequential use of instruments is safe	Yes	24 (17.3%)
	No	72 (51.8%)
	Unsure	43 (30.9%)
If there is no progress following three traction attempts, caesarean section should be considered	Yes	121 (87.1%)
	No	5 (3.6%)
	Unsure	13 (9.4%)
Rotational forceps has been abandoned	Yes	67 (48.2%)
	No	26 (18.7%)
	Unsure	46 (33.1%)
ventouse delivery has a lower success rate than forceps delivery	Yes	42 (30.2%)
	No	53 (38.1%)
	Unsure	44 (31.7%)
ventouse delivery is safer than forceps delivery	Yes	68 (48.9%)
	No	34 (24.5%)
	Unsure	37 (26.6%)

Eighty-nine (64%) of the obstetricians declined encouraging ID if in a situation that indicated the use of ID.

Possible reasons for abandoning ID included reduction of training (116/139, 83.5%), lack of senior supervision (99/139, 71.2%), fear of litigation (125/139, 89.9%), their convenience that emergency CS in the second stage is safer than ID (86/139, 61.9%), lack of consultant supervision in acute situations (108/139, 77.7%), lack of modern equipment (108/139, 77.7%), and financial gains with performing CS (77/139, 55.4%) (Table 5).

**Table 5:** Possible reasons for not recommending ID

A reduction in training	Yes	116 (83.5%)
	No	23 (16.5%)
Lack of senior supervision	Yes	99 (71.2%)
	No	40 (28.8%)
Fear of litigation	Yes	125 (89.9%)
	No	14 (10.1%)
Emergency caesarean section in the second stage is safer than instrumental vaginal delivery	Yes	86 (61.9%)
	No	53 (38.1%)
Instrumental vaginal delivery is not associated with spontaneous vaginal delivery in a subsequent pregnancy	Yes	46 (33.1%)
	No	93 (66.9%)
Consultant supervision and teaching is not always possible in acute situations	Yes	108 (77.7%)
	No	31 (22.3%)
It is not recommended by the ministry of health	Yes	74 (53.2%)
	No	65 (46.8%)
Lack of modern equipment	Yes	108 (77.7%)
	No	31 (22.3%)
Financial gains may accompany the performance of CS	Yes	77 (55.4%)
	No	62 (44.6%)

## Discussion

A great proportion of the obstetricians never tried ID before. Even those who practiced ID had less experience, although it is documented by the WHO as one of six fundamental skills in critical obstetric care [1]. A recent report mentioned that ID was the least performed skill in basic institutions [3]. Other countries reported lack of ID education in many medical schools [8].

A great proportion of the physicians had a neutral attitude towards encouraging ID, although agreeing to present it as an option. More than half of the obstetricians declined encouraging ID if in a situation that indicated the use of ID. An earlier study evaluating the national attitudes of physicians towards vacuum delivery in 121 developing countries, and reported varied rates of ID. In Latin America, 74% of the countries never used ID. In Sub-Saharan Africa and Asia, 40% and 30% of countries, respectively never used ID [15]. Another report declared refusal of a great number of Egyptian obstetricians to perform any procedure that might decrease the CS rate as ID [5].

Possible reasons for abandoning ID included reduction of training, lack of senior supervision, fear of litigation, their convenience that emergency CS in the second stage is safer than ID, lack of consultant supervision in acute situations, lack of modern equipment, and financial gains with performing CS.

### Limited resources

A previous report mentioned outdated equipment as a main finding when analysis possible causes for disappearing ID art [6]. Other causes for low ID especially in low resource countries were increased parity, lack of epidural analgesia, and lack of electronic fetal monitoring [16].

### Deficient training

Other contributing factors were lack of training, reluctance of trained obstetricians to perform ID, lack of endorsement in institutional policies, and financial gains with performing CS [8]. A recent study reported one of the reasons for declined ID rates was lack of ID training in residency training programs [17].

### Easily accessible and harmless CS

Other causes included increased accessibility of CS in the second stage [12]. There is no evidence that CS would improve maternal or fetal outcomes. Extremely elevated rates of CS are unjustified [18]. Another study reported refusal of ID by the obstetricians and pediatricians because of its possible fetal harm [12].

### Other contributing factors

Other reasons included the absence of national or institutional guidelines in the Egyptian healthcare sector [19]. In addition, antenatal care programs do not include teaching classes about ID [20]. Also, increased financial gains with CS than vaginal delivery played a paramount role in the declined rates of ID [21].

### Strengths and limitations

Scarce studies reported on obstetricians’ attitude towards ID in Egypt. This study elaborated possible causes for abandoning ID among Egyptian obstetricians. However; the sample size is a limitation. It has been estimated that a sample size of about 300 participants would be representative for a population of about 6000 for categorical data [22]. Unfortunately, we do not have an accurate estimate for the number of obstetricians in Egypt, with approximated estimates of about 6700 obstetricians in the governmental sector only. Accordingly, this sample size would limit the generalizability of the results.

### Research implications

ID in Egypt is considered a vanished art, accompanied with a tremendous increase in CS rates. Proper training of the obstetricians is a must. Setting definitive institutional guidelines concerned with ID would help in decreasing CS rates and encouraging the obstetricians to practice ID when indicated.

## Conclusions

ID is not widely practiced in Egypt. Great effort should be directed towards increased physicians’ awareness and experience regarding ID, although the chances for training would be in emergency situations, limiting opportunities for training.

## Data Availability

The datasets used and/or analyzed during the current study are available from the corresponding author upon reasonable request. All data generated or analyzed during this study are included in this published article.

## Abbreviations

ID: Instrumental delivery
WHO: World health organization
CS: Cesarean section
